# Factors associated with delay in seeking treatment among women with pelvic organ prolapse at selected general and referral hospitals of Southern Ethiopia, 2020

**DOI:** 10.1186/s12905-021-01245-0

**Published:** 2021-03-01

**Authors:** Asfaw Borsamo, Mohammed Oumer, Yared Asmare, Ayanaw Worku

**Affiliations:** 1 Department of Human Anatomy, College of Medicine and Health Sciences, Hawassa University, Hawassa, Ethiopia; 2 Department of Human Anatomy, School of Medicine, College of Medicine and Health Sciences, University of Gondar, Gondar, Ethiopia; 3 Department of Epidemiology, Institute of Public Health, College of Medicine and Health Sciences, University of Gondar, Gondar, Ethiopia

**Keywords:** Pelvic organ prolapse, Delay in seeking treatment, Ethiopia

## Abstract

**Background:**

Pelvic organ prolapse (POP) is the descent of the vaginal wall, cervix, uterus, bladder, and rectum downward into the vaginal canal. Its prevalence is higher among women in developing countries because women are more prone to risk factors. In Ethiopia, women with prolapse seek treatments at advanced stages of prolapse; hence, surgical management has been widely practicing. Therefore, it was found to be very important to conduct research that assesses factors hindering early treatments in Southern Ethiopia. This study aimed to find out factors associated with the delay in seeking treatment of pelvic organ prolapse among patients at selected general and referral hospitals of Southern Ethiopia.

**Methods:**

Cross-sectional study design was employed in 123 participants of seven randomly selected General and Referral Hospitals of Southern Ethiopia from February 01 to April 30, 2020, by using a structured questionnaire. Pre-trained two midwives in each center were deployed to collect data. Physicians performed diagnosis and physical examination. Data were entered and coded using EPI INFO version 7 and exported into SPSS version 25 for analysis. Bivariate and multivariable logistic regression analyses were performed. The goodness of fit was assessed by using the Hosmer and Lemeshow goodness test.

**Results:**

In this study, out of 123 clinically diagnosed POP cases, nearly half of them were stage III, and over one-third were stage IV. Therefore, 84.6% (104 participants) of the respondents were delayed for the treatment of POP. The mean length of delay for POP treatments was 36.41 ± 3.95 months. After adjusting for covariates, lack of supports [AOR (Adjusted Odds Ratio) = 5.2 (95% CI 1.4–19.5)], low-income [AOR = 5.8 (95% CI 1.1–19.66)], and fear of social stigma [AOR = 4.7 (95% CI 1.2–18.59)] were significant factors for delayed treatments.

**Conclusions:**

Most of the POP patients were delayed for POP treatments. Factors like lack of support, low-income, and fear of losing social value/stigma were associated with treatment delay. Screening for the POP cases, educating (making awareness) the community about this devastating disease to facilitate early treatment and to avoid social stigma, and raising access to treatment by making the nearby hospitals equipped with facilities to treat POP are recommended.

## Background

Pelvic organ prolapse (POP) is the herniation of the uterus, the cervix, the vaginal wall, the bladder, and the rectum downward along the vaginal lumen [1]. It is a common gynecological problem, which is more prevalent in developing countries, that affect the life quality of women by limiting emotional, physical, psychosocial, and sexual functions [2, 3].

The prevalence of POP varies widely from population to population depending on the level of exposure to the risk, level of awareness about POP, and level of the socio-economic status. Universally, the prevalence of this problem is about 3.5% to 15% when it is studied by defining symptoms and it reaches up to 64.6% when studied based on objective findings, which are directly measured by the experts [3–8].

Women in the developed countries who have access to modern health care can benefit from the advances that have been made in treating POP. Moreover, women in developed nations have an awareness of the problem, high literacy rate, and high-income level with excellent health facilities that can screen the POP very early. Delaying for treatment is also affected by high parity, little or no access to health care, and the presence of problems associated with POP [9].

The prevalence of POP in Africa varies widely from country to country: 64.6% in Tanzania [5], 19.4% in Egypt [10], and 6.5% in Nigeria [11]. The prevalence of the POP in Ethiopia is also highly variable ranging from 1 to 56.4% [3, 7, 12]. For instance, 40.7% among gynecologic case in Jimma Hospitals [13], 15% among gynecologic case in Saint Paulos Hospital, Addis Abeba [14], 56.4% in Dabat community, Gondar [3], 20.9% in Kersa community, Ethiopia [7], and 13.3% in Benchi Maji Zone [15].

Essentially, delaying the POP treatments worsens the problem further in Ethiopia. Many women are silent victims of uterovaginal prolapse, hiding the problem owing to fearing social stigma. Despite uterovaginal prolapse is a matter of discomfort for women, it affects many aspects of their daily life [12, 16]. According to a community-based objectively measured study at Dabat district, Gondar, the prevalence of POP is about 56.4%, however, only patients with advanced stages of POP (3.2%) visited the hospitals, even if free service is providing [3].

According to few studies assessing reasons for delayed treatments among women within Ethiopia, the possible reasons are lack of support, low awareness, low literacy, unavailability of the treatment center, low income, and disclosure of the problem or fear of social stigma [12, 16]. In Ethiopia, although the non-governmental organizations in collaboration with the Federal Ministry of Health tried to deliver free services for the victims integrating with obstetric fistula care, the women kept home hiding their problems even from their close partners. This indicates that little attention is given to health education, and creating awareness on the problem. Besides, few studies were conducted on reasons hindering early treatment [12, 16].

There is a scarcity of the study assessing factors associated with the delay in seeking treatment among women with POP in Southern Ethiopia. Notably, exposing factors that hinder early treatments of POP initiates up responsible bodies to take interventional actions at the early stages of the problem. Therefore, it was found to be very important to conduct research that assesses factors associated with the delay in seeking treatment among women with POP in South Nations Nationalities and Peoples’ Region (SNNPR).

## Methods

### Study design and setting

The hospital-based cross-sectional study design was conducted from February 01 to April 30, 2020, in seven (out of thirteen hospitals) randomly selected governmental general and referral hospitals of southern Ethiopia. All gynecologic patients diagnosed with a POP at selected governmental general and referral hospitals of Southern Ethiopia were included in the study. A total of 123 eligible POP women participated in the study.

### Measurement

POP was evaluated and described using a standardized Pelvic Organ Prolapse Quantitative Examination Tool. In this technique, the hymen ring (remnant) is considered as a reference point [1, 17]. The stages of POP were presented in Table 1.

**Table 1:** The pelvic organ prolapse staging, 2020

Stages	Description
0	No prolapse [1, 17]
I	The leading-edge of the prolapse is 1 cm above the level of the hymen (> − 1 cm) [1, 17]
II	The leading-edge of the prolapse lies through the plane of hymen between − 1 cm and + 1 cm [1, 17]
III	The most distal portion of the prolapse is between + 1 cm and + 2 cm below hymen [1, 17]
IV	The complete eversion of the total length of the pelvic organ (> + 2 cm)

### Operational definitions

*Delayed treatment of POP:* Advanced stage prolapses treated with surgical management regardless of the duration of the onset of symptoms [1]. *Early treatment of POP:* Early-stage prolapses treated with conservative management regardless of the duration of the onset of symptoms [1]. *Low income:* Those who earn less than 1200 Birr monthly were considered as low income and, practically, those women who have the interest to go hospital but delay in seeking treatment due to the lack of money [12, 18]. *Lack of support:* Those women who delayed in seeking treatments because they do not have a person who supports them financially and emotionally [12, 16]. *Culture influence:* In some cultures in Ethiopia, it is extremely shameful exposing, discussing, and talking about anything related to genitalia (sensitive body parts), even to their daughters and children [12]. *Being busy:* Those women who delayed in seeking treatments because they are busy with works and with different responsibilities [19]. *Fear of social stigma*: A woman with POP who delayed in seeking treatments due to the high concern of social discrimination [11, 12].

### Data collection tools, techniques, and procedures

Data were collected using the interviewer-guided structured questionnaire, having two parts: The first part assessed objective findings, and the second part assessed factors for delayed treatments. To assess objective findings, physicians first took the surgical and medical history of the patients. Then, they performed vaginal examinations and diagnoses. Based on Standardized Pelvic Organ Prolapse Quantitative Examination Tool, physicians determined the stages of POP. After the physician decided on management, participants were interviewed.

The questionnaire was developed by the investigators by reviewing different related literature [3, 11, 12, 16, 20]. Then, the questionnaire was evaluated by the experts in the profession to ensure validity. The questionnaire was first prepared in English, and, then, appropriately translated into the Amharic language. Later, translated back to English to ensure the accuracy of the meaning. A pretest was carried out on 10% of the sample size at the Shashemene General Hospital, Oromia, Ethiopia, and Bushulo General Hospital, Sidama, Ethiopia (private obstetrics and gynecology center) mainly to check the general approachability, validity, and feasibility of the questionnaire. As a result, corrections and modifications were made to the questionnaire accordingly. Two midwives collected the data using the Amharic version questionnaire under strict supervision.

### Data quality control and management

Data quality was controlled through the provision of one-day training to the data collectors about the overall objective of the study, the definition of terms and concepts, approaching of respondents, data collection tools, and techniques of interviewing. The collected data were also cross-checked for its completeness, consistency, accuracy, and clarity daily. The investigators carried out close site supervision during the whole data collection period to monitor overall data collection quality.

### Data processing and analysis

After data collection, each questionnaire was again visually checked for completeness, clarity, and accuracy. Data were coded and entered into EPI INFO version 7 software and exported to SPSS version 25 statistical software for analysis. Descriptive, bivariate, and multivariable logistic regression analyses were performed. Selected variables that have a P value of ≤ 0.2 at the bivariate logistic regression analysis were included in the multivariable logistic regression to control all possible confounding factors simultaneously. The goodness of fit was assessed by using the Hosmer and Lemeshow goodness test. Generally, a P value ≤ of 0.05 was considered statistically significant.

## Results

### Socio-demographic characteristics of the participants

Out of 123 participants, the majority (61%) were aged ≥ 45 years. Over two-thirds (67.48%) of the participants did not attend formal school at all. About 66.70% of participants were rural residents and 71.54% were housewives. The majority (69.01%) earns ≤ 1200 Ethiopian birrs monthly (Table 2).

**Table 2:** Sociodemographic characteristics of the participants at selected general and referral hospitals of Southern Ethiopia, 2020

Variables	Frequency (n = 123)	Percent (%)
Age		
≤ 35	13	10.57
34–44	35	28.46
≥ 45	75	60.97
Ethnicity		
Wolaita	33	26.83
Sidama	27	21.95
Gurage	16	13.00
Hadiya	14	11.38
Amhara	8	6.50
Oromo	8	6.50
Kambata	7	5.70
Gamo	6	4.88
Dorze	2	1.63
Amaro	1	0.81
Halaba	1	0.81
Religion		
Protestant	68	55.28
Orthodox	43	34.96
Muslim	9	7.32
Waka and traditional believers	7	5.69
Residency		
Rural	82	66.70
Urban	41	33.30
Occupational status		
House wife	88	71.54
Farmer	23	18.70
Merchant	23	18.70
Employed	24	19.51
Educational status		
No schooling	83	67.48
Primary	18	14.63
Secondary	15	12.20
Diploma +	7	5.69
Marital status		
Married	104	84.55
Widowed	10	8.13
Divorced	9	7.32
Monthly income (ETB)		
> 1200	38	30.89
≤ 1200	85	69.01

### Description of delayed treatments

One hundred twenty-three cases were clinically diagnosed for POP. Out of 123 POP cases, nearly half (60 participants) of them were stage III, and over one-third (44 participants) were stage IV. Therefore, 84.6% (104 participants) of the respondents were delayed for the treatment of POP. The mean length of delay for POP treatments was 36.41 ± 3.95 months (Table 3).

**Table 3:** Summary of stages of POP and management types at selected General and Referral Hospitals of Southern Ethiopia, 2020

Stage of POP	Frequency	Percentage (%)
Stage I	0	0
Stage II	19	15.4
Stage III	60	48.8
Stage IV	44	35.8
Management types		
Surgically managed	104	84.6
Conservatively managed	19	15.4

### Reasons for delayed treatments

Out of 123 women with POP, 58.54% of women complained of a lack of support as the reason for the delayed treatments. The majority (73.17%) reasoned out a lack of transportation for delayed treatments (Table 4).

**Table 4:** Reasons for delayed POP treatments among participants at selected General and Referral Hospitals of Southern Ethiopia, 2020

Variables	Delayed treatment (n = 104)	Early treatment (n = 19)	Total (n = 123)	Percent (%)
Lack of support				
No	38	13	51	41.46
Yes	66	6	72	58.54
Low income				
No	49	15	64	52.03
Yes	55	4	59	47.97
Lack of transportation				
No	19	14	33	26.83
Yes	85	5	90	73.17
Unavailability of treatment centers				
No	75	16	91	78.98
Yes	29	3	32	21.02
Believing POP as normal				
No	70	18	88	71.54
Yes	34	1	35	28.46
Fear of surgery				
No	53	12	65	52.84
Yes	51	7	58	47.16
Culture influence				
No	58	13	71	57.72
Yes	46	6	52	42.18
Fear of stigma				
No	40	14	54	43.90
Yes	64	5	69	46.10
Fear of disclosure				
No	42	14	56	45.53
Yes	62	5	67	54.47
Being busy				
No	62	11	73	59.34
Yes	42	8	50	40.66

### Factors associated with delayed treatments

On bivariate logistic regression analysis, lack of support, low-income, lack of transportation and road, believing POP as normal, fear of losing social value/stigma, fear of disclosure, maternal age, and educational status were significant reasons for delayed treatments. On multivariable logistic regression analysis, lack of support, low-income, and fear of losing social value/stigma were reasons associated with delayed treatments of POP patients. Accordingly, women with a lack of support were five times [AOR = 5.2 (95% CI 1.42–19.5)] more likely to delay treatments of POP than their counterparts. Women with low-income were nearly six times [AOR = 5.8 (95% CI 1.1–19.66)] more likely to delay treatments of POP than women with higher income. Women who fear social stigma was about nearly five times [AOR = 4.7 (95% CI 1.2–18.59)] more likely to delay treatments as compared to their counterparts (Table 5). The value of the Hosmer and Lemeshow goodness of fit test was 0.332.

**Table 5:** Logistic regression analysis about factors associated with the delay in seeking treatment of POP patients at selected general and referral Hospitals of Southern Ethiopia, 2020

Variables	Delayed treatments	Early treatment	COR(95%CI)	AOR (95%CI)
Lack of support				
No	38 (74.5%)	13 (25.5%)	1	1
Yes	66 (91.7%)	6 (8.3%)	5.2 (2.0–15.36)	5.2 (1.42–19.5)*
Low income				
No	49 (76.6%)	15 (23.4%)	1	1
Yes	55 (93.2%)	4 (6.8%)	4.2 (1.3–13.54)	5.8 (1.1–19.66)*
Lack of transportation				
No	19 (57.6%)	14 (42.4%)	1	1
Yes	85 (94.4%)	5 (5.6%)	8 (2.628–24.26)	2.2 (0.56–8.48)
Unavailability of treatment centers				
No	75 (82.45)	16 (17.6%)	1	
Yes	29 (90.6%)	3 (9.4%)	2.06 (0.56–7.6)	–
Believing POP as normal				
No	70 (79.5%)	18 (20.5%)	1	1
Yes	34 (97.1)	1 (2.9%)	8.74 (1.12–68.2)	9.9 (0.96–11.3)
Fear of surgery				
No	53 (81.5%)	12 (18.5%)	1	1
Yes	51 (87.9%)	7 (12.1%)	1.65 (0.6–4.52)	–
Culture influence				
No	58 (81.7%)	13 (18.3%)	1	
Yes	46 (88.5%)	6 (11.5%)	1.7 (0.61–4.87)	–
Fear of social stigma				
No	40 (74.1%)	14 (25.9%)	1	1
Yes	64 (92.8)	5 (7.2%)	4.48 (1.5–13.39)	4.7 (1.2–18.59)*
Fear of disclosure				
No	42 (75%)	14 (25%)	1	1
Yes	62 (92.5%)	5 (7.5%)	4.1 (1.4–12.34)	4 (0.99–16.43)
Being busy				
No	62 (84.9%)	11 (15.1%)	1	
Yes	42 (84%)	8 (16%)	0.93 (0.35–2.51)	–

Key: AOR, adjusted odds ratios; COR, crude odds ratios

^*^Statistically significant at P value ≤ 0.05 in multivariable logistic regression analysis

## Discussion

This study primarily assessed factors associated with the delay in seeking treatment among women with POP at selected general and referral hospitals of Southern Ethiopia. Accordingly, lack of support, low income, and fear of social stigma were significant factors associated with delayed treatments of POP patients. This study also assessed reasons for delayed treatments among POP patients at selected general and referral hospitals of Southern Ethiopia. Out of 123 POP patients, only 15.4% of POP patients came with earlier stages of POP. The mean length of delay for POP treatments was 36.41 ± 3.95 months. This finding is comparable with the study finding of Israel (43 months) [21]. Nevertheless, it is lower when compared with the mean length of delay in the study conducted in Gondar (85.8 ± 80.2 months) [12]. The most likely justification for this discrepancy is that in our study the patients with POP visited the hospital for seeking treatments by themselves. Meanwhile, in the Gondar study, there was a community-based screening and campaign for searching the cases of POP and, in this case, a lot of cases of POP may surveyed for the treatments and several cases with longer duration may contribute to the high value of estimates because in the community we can find a woman who stayed for longer period by hiding the problem.

This study revealed women with a lack of support were over five times more likely to delay treatments of POP than their counterparts. This is inconsistent with the report from the study of Gondar saying lack of support is not associated with delaying for the treatments [12]. The possible justification for this discrepancy is most probably the POP care in Gondar is integrated into the fistula care center. In Gondar, women with POP are free of any payment related to POP treatments including transportation.

In this study, low income was significantly associated with delayed POP treatments. This is supported by the study conducted in Gondar, which reported the women with a lack of money were about two times more probably delay POP treatments (AOR = 2, 95% CI 1.01, 3.86) [12]. Women with a shortage of income were not able to afford the health service expenses, transport, food, and other costs [22, 23]. It is a fact that most rural mothers in Ethiopia are not economically empowered [23]. Besides, in our context, women have many responsibilities that should carry out in day-to-day activities but not to generate income, and the power to make the financial decision is not by their hands and these and thus situation doubles their problems [23].

In this study, fear of social stigma increases the likelihood to delay treatments. A study conducted to assess reasons for delayed treatments of POP and fistula patients at the University of Gondar Comprehensive and Specialized Hospital, Ethiopia, reported fearing social stigma was not associated with delay for treatments of POP. However, that study reported women who fear disclosing fistula owing to social stigma were two times more likely to delay the fistula treatments when compared with their counterparts [12]. The possible justification for the discrepancy is most probable Hamlin Fistula Center in Gondar has been delivering integrated service of fistula and POP care, repeatedly conducting the POP campaign and expanding awareness creation about the problem in the Gondar community.

In this study, out of 123 POP patients, 7.3% were divorced due to POP. This is comparable with the report of a study from Uganda that reported their husband rejected about 25% of women with POP [20]. The same was true in Nepal among women with POP [24] and in Gondar among women with fistula [12]. Moreover, qualitative studies in Nepal and Bahr Dar also believe that social stigma is the most important barrier to seek health care and to expose problems even to their close ones feeling it shameful because POP involves the most sensitive part of the body (genitalia) [16, 24]. Furthermore, due to fear of discrimination and rejection from people, they hide the illness at all and they disclose when it gets severe and unbearable after an extended duration of time since they begin to feel symptoms. Because of this delay, the problem reaches its advanced stage, which causes surgical interventions to become the only possible treatment solution. Notably, most women in Southern Ethiopia have less awareness about the POP and unable to reveal their problems related to their genitalia (sensitive body parts) because of the feeling of shamefulness, which has a deep root in the culture of the society.

As a limitation, this study was hospital-based and, hence, lacks generalizability to the community at large. Moreover, further study is needed in different places and the population.

## Conclusions

In this study, most of the POP patients were delayed for POP treatments. Factors like lack of support, low income, and fear of losing social value/stigma were associated with the delay in seeking treatment. Screening for the POP cases, educating (making awareness) the community about this devastating disease to facilitate early treatment and to avoid social stigma, and raising access to treatment by making the nearby hospitals equipped with facilities to treat POP are recommended.

## Data Availability

The datasets used and/or analyzed during the current study are available from the corresponding author on reasonable request.

## Abbreviations

AOR: Adjusted odds ratios
COR: Crude odds ratios
POP: Pelvic organ prolapse
SNNPR: South Nations Nationalities and Peoples’ Region
